# Exploring Barriers to Vitamin A Supplementation Uptake and Program Implementation Among Children Aged 6–59 Months in Ethiopia: A Qualitative Approach

**DOI:** 10.3389/ijph.2023.1606167

**Published:** 2023-09-29

**Authors:** Getahun Fentaw Mulaw, Seteamlak Adane Masresha, Fentaw Wassie Feleke

**Affiliations:** Department of Public Health, College of Health Sciences, Woldia University, Woldia, Ethiopia

**Keywords:** children, vitamin A supplementation, qualitative approach, Amhara, Ethiopia

## Abstract

**Background:** Children in Ethiopia do not receive the recommended dose of vitamin A supplementation (VAS).

**Objective:** This study aimed to explore the barriers to VAS uptake and program implementation among children aged 6–59 months in Ethiopia.

**Methods:** Data were collected qualitatively using focus group discussions and in-depth interviews. The data were audio-recorded, transcribed, and inductively coded. The results were displayed using thematic analysis and the well-spoken verbatim of the respondents.

**Results:** The barriers to VAS uptake were low parental awareness of the program and low interest or motivation, low promotion of the program among health professionals, an excessive workload for health extension workers, and low professional attention to VAS compared to other health services.

**Conclusion:** Both client-side and supply-side issues posed significant obstacles to the uptake of VAS. It is important to educate mothers about VAS. Health workers should receive refresher training to raise their level of concern about VAS’s importance and its schedule. It’s crucial to initiate outreach programs for remote communities. The departmentalization of health extension packages will improve service and access.

## Introduction

Around two billion people globally suffer from micronutrient malnutrition [1]. The prevalence of vitamin A deficiency (VAD) among children aged 6–59 months was one-third worldwide in 2013, with sub-Saharan Africa (SSA) having one of the highest rates (48%) of VAD [2]. In 44 of the 49 SSA nations, VAD has a significant impact on public health [3]. Furthermore, VAD is responsible for about 2% of all pediatric fatalities under the age of five [4]. It primarily impacts 130 million preschoolers in developing nations [3] and raises their mortality risk by 20%–30% [5]. Children with VAD have a depressed immune system, which makes them more susceptible to infections and increases their risk of developing common childhood illnesses like respiratory illnesses, diarrhea, measles, and eye issues [4, 6–8].

VAD causes Xerophthalmia, a variety of eye conditions ranging from night blindness to more severe clinical outcomes like keratomalacia and corneal scars and permanent blindness [9]. There are approximately 5 million preschool-aged cases of xerophthalmia, 10% of which have the potential to cause blindness [10]. In Ethiopia, the prevalence rates of Bitot’s spots and night blindness among children are 1.7% and 0.8%, respectively, and the prevalence of subclinical deficiency is 37.7%. Thirty-two percent of child deaths in Ethiopia are attributable to VAD [11, 12].

Improving the vitamin A status of children will play a critical role in reducing child mortality [13, 14]. To increase child survival, it is necessary to guarantee universal VAS coverage (100%) or at the very least efficient coverage (80%) [15]. In 2016, only 64% of children in need in priority countries were reached with two doses of VAS but more than 140 million children were left behind, leaving them vulnerable to disease and death. In SSA, which has the highest infant mortality rate in the world (78 deaths per 1,000 live births), VAS coverage is declining. In 2016, only 10 SSA’s had effective coverage [15].

Currently, VAS is administered routinely in Ethiopia as part of the Health extension program (HEP) to raise and maintain VAS coverage among under-five children above 80% [16–20]. Even though numerous initiatives to expand VAS coverage have been launched, their execution still falls short of expectations. According to the Ethiopian Demographic health survey (EDHS) 2019, the national VAS coverage among children aged 6–59 months was 47.1%. In Amhara region it is 58.4% [21]. Additionally, specific area studies conducted across Ethiopia have revealed that children aged 6–59 months have a low VAS coverage rate. For instance, a study done in Libo kemkem and Fogera district, Amhara region showed that only 29.3% of children received VAS [22].

Therefore, this study was aimed at exploring the barriers to VAS uptake and programme implementation among children aged 6–59 months in Ethiopia. This would be beneficial for developing new VAS coverage strategies and reviewing existing ones for health policy makers, programme administrators, service providers, and other stakeholder groups.

## Methods

### Study Design, Area, and Period

A qualitative explanatory research design was conducted in the North-Wello zone from 1 March 2021 to 15 March 2021. The North-Wello zone is found 520 km northeast of Addis Ababa, the capital city of Ethiopia, and 360 km north of Bahir Dar, the capital city of the Amhara region. North-Wello is one of the zones of the Amhara Region, having 11 districts. According to the projection of the 2007 Ethiopian Census, the total population of the zone is estimated to be 1,788,901, of which children under the age of five constitute 241,502. Within the zone, there are five general hospitals, 65 health centers, and 306 functional health posts. The people of the district are mainly an agrarian community**.**

### Study participants

The study participants were mothers who had given birth to at least one child within the previous 5 years, women’s development armies, health professionals, health centres, and woreda health office heads.

### Sample Size and Sampling Technique

The sampling adequacy was determined by the point of idea saturation, despite the fact that there is no established and unanimous way of determining sample size for qualitative studies [23, 24]. Participants were selected using purposeful sampling, which took into consideration a range of sociodemographic factors (such as age, education, place of residence, and religion), a range of health workers working at various levels, and a range of educational backgrounds. Participants for the Focus Group discussion (FGD) and in-depth interview were selected from four districts with the assistance of local coordinators. A total of four focus-group discussions, comprising a total of 38 participants (8–12 members in each group), and 12 in-depth interviews were conducted at the Woreda and Kebele labels.

### Data Collection Procedures and Data Quality Control

The data were collected through FGDs and in-depth interviews. Two unstructured interview guides for in-depth interviews (one customized to each subgroup of participants) and one unstructured interview guide for focus group discussions were created and translated into the local language, Amharic. The interview guides shared a base of common questions and included additional questions based on the participant’s status. Each interviewer followed an open-ended guide to assess the level of knowledge and awareness about VAS, the barriers to its implementation, and which scheme (routine delivery or Enhanced outreach strategy) is important to increase coverage. Each in-depth interview took 30–60 min, and each FGDs took 60–90 min. The data were collected by the research teams with the support of local coordinators. All interviews and focus groups were audio recorded, aided by key-note keeping.

The location or spot for FGD and in-depth interviews was chosen while taking care to ensure participant comfort, convenience, and minimal destruction. A double audio recording was made, and careful handling was used when taking some critical notes.

### Data Management, Processing, and Analysis

The tape-recorded data were transcribed verbatim and translated into English. Then the interviewer’s extensive notes were reviewed. First, each FGD and in-depth interview were thoroughly read and re-read to develop a coding scheme. Then, initial line-by-line coding was done to identify relevant themes. In the next step, selective coding was performed, and relevant codes were further categorized to form themes. Finally, the data was analyzed inductively using thematic analysis. Verbatim quotations were frequently used to illustrate the responses of the respondents to important issues and subthemes.

### Ethical Consideration

The study protocol was reviewed and approved by the Ethical Review Committee of Woldia University, College of Health Sciences (ERC0045/2021). An official letter of support was written to the North-Wello Health Bureau and respective woredas health offices to seek their essential cooperation. Before starting the data collection process, the objective, purpose, possible procedures, potential risks and benefits of the study, and the possibility of withdrawing from the FGDs and interview were explained to participants to obtain informed verbal consent.

The participants were assured of the confidentiality of the data. No personally identifiable information was collected on the data collection tools, and instead of using names, codes were given to call the participants during the interviews and FGDs. Any identifiers included in audio recordings were removed during transcription. All audio recordings were permanently deleted after full transcription. Finally, after each interview and FGD, the data collectors and research teams communicated the importance and schedule of VAS and other specific interventions (deworming and dietary feeding practice) based on the responses of mothers.

## Results

### Socio-Demographic Characteristics of Participants

A total of 38 mothers participated in four focus group discussions of whom 13 are in the age range of 18–25 years and 12 are in the age range of 26–35 years. More than half [18] of the mothers were from rural communities (Table 1).

**TABLE 1:** Socio-demographic characteristics of focus group discussion participants, to assess the barriers for vitamin A supplementation uptake among children aged 6–59 months in the North-Wello Zone, Ethiopia, 2021.

Variable	Category	Frequency (n = 38)
Mothers age	<18 years	2
	18–25	13
	26–35	12
	>35	11
Education	Not educated	14
	Primary	11
	Secondary and above	13
Residence	Rural	23
	Urban	15
Religion	Orthodox	26
	Muslim	12
Occupation	Housewife	14
	Farmer	11
	Private small business	7
	Employed	6

Participants in the in-depth interviews, comprising of women’s development army members, health extension workers, health professionals working in health centers, nutrition focal persons, health center heads, district health office heads, and mothers, were conducted (Table 2).

**TABLE 2:** Socio-demographic characteristics of in-depth interview participants to assess the barriers for vitamin A supplementation uptake among children aged 6–59 months in the North-Wello Zone, Ethiopia, 2021.

Age	Sex	Educational level	Occupation (role)	Residence	Marital status
38	F	Not educated	Farmer	Rural	Married
30	F	Primary	Housewife	Rural	Married
30	F	Primary	Women development army	Rural	Married
33	F	Primary	Women development army	Rural	Divorced
38	M	BSc degree	Woreda health department head	Urban	Widowed
37	M	BSC degree	Health center head	Urban	Married
24	F	Diploma	Health extension	Rural	Single
28	F	Diploma	Health extension	Rural	Married
30	F	Diploma	Nutrition focal (Public Health)	Rural	Married
41	M	BSc Nurse	Child health and Nutrition officer	Urban	Married
31	M	BSC degree	Health officer (working in under five outpatient department)	Urban	Married
29	F	BSC degree	Clinical nurse working in under-five outpatient department	Urban	Divorced

### Barriers to Uptake of VAS and Program Implementation

**FIGURE 1:**
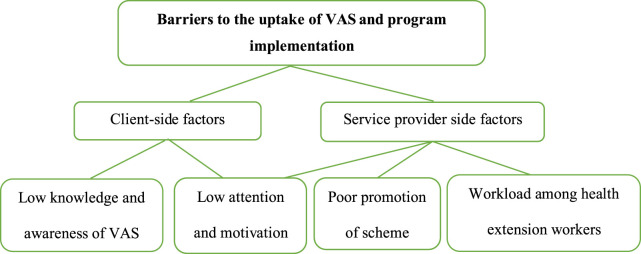
Themes and categories created during the qualitative analysis for identifying the barriers to vitamin A supplementation uptake among children aged 6–59 months in the North-Wello Zone, Amhara Region, Ethiopia, 2021.

During interview and FGD’s a number of barriers for VAS uptake and program implementation were identified. These barriers were inductively coded in to into two broad themes and further five categories. The two broad themes are client level barriers and service provider level barriers (Figure 1). Some of the example(s) that best explain the category were selected from respondents’ well-spoken verbatim during FGD and key informant interviews. These quotes are presented in italics and between quotation marks.

### Low Knowledge and Awareness of the Importance of VAS and Its Schedule Among Parents

The data indicated that most parents did not know about the importance of VAS, the consequences of vitamin A deficiency, or the schedule of VAS. Though some mothers have heard about VAS, they don’t know the definite schedule of VAS.

A 22 year-old urban resident mother replied to the question, “Do you know the importance of VAS and when the child should take it?”

“…Leave the importance and the schedule, totally. I did not hear of the existence of such a service (to mean a VAS program) for children. Always when I go to a health center or health post, I get counseling on the child immunization schedule but not on the VAS.”

A 29 year-old mother also stated that

“…*Since we do have access to vegetables and fruits, I believe that my child could get Vitamin A from those food items. Rather, I give attention to my child not to miss the vaccination schedule.”*

A 33 year-old woman in the development army also argued that

“…As a woman in the development army, I should have to know about all the services delivered by health posts. But until today, I didn’t have the information. Most mothers have not enough knowledge on the importance and schedule of VAS.”

### Parental and Health Professionals’ Low Attention and Motivation

The data also showed that mothers and health extension workers place less emphasis on VAS, especially for older children above the age of two. They only concentrate on the vaccination schedule, and mothers are also inclined to get Plumy-Net for their children.

Another 37 year-old woman also replied as

“…Most of the time we went to health posts eagerly to receive Plumpy-Nut; even though the health extension workers gave us information on other health services, our attention was on getting the Plumpy-Nut. We do not give equal weight to other services, even immunization.”

A 28 year-old health extension worker said that

“…There is low motivation among mothers on VAS. Mothers did not bring their children every six months to health posts or health centers; more of those who traveled long distances did so. For those living far away from health posts, the regular house-to-house delivery was better to reach them.”

A 32 year-old mother also commented that

“…. I know about when it is taken, but I did not give emphasis on it and did not believe it could protect against diseases. Rather, I believe in the importance of child vaccination.”

A 38 year-old degree holder and head of the health department summed up the barriers for VAS uptake as follows:

“…Even though the accessibility is good, all children did not receive it because of various conditions. There is low motivation by mothers and even health extension providers, and the health extension workers by themselves do not believe its importance is equal to that of the vaccination schedule.”

A 31 year-old public health officer professional working in the under-five OPD argued that

“…Currently, most health professionals do not give emphasis to VAS. Even my children, aged 18 months, did not take VAS. We, as health professionals, did not give attention to VAS during our counseling session. I think refreshment trainings and refocusing on VAS programs to reach the most vulnerable are needed.”

A 37 year-old degree-holder health center head and a 24 year-old health extension worker, both argued that

“…The former door-to-door campaign was better at reaching children than the current routine delivery mode. Because the campaigns were easy to mobilize the community and raise mass awareness during each campaign session. It also will increase coverage of older children, because during routine follow-up, mothers only bring their younger children to the health posts; they do not consider it vital for the older ones, too.”

### Low Promotion of the Scheme by Health Professionals

Some parents argued that there is no or little promotion of the scheme by health professionals, unlike that of vaccination, institutional delivery, and the feeding practice of sick children. Even the health professionals themselves believe that there is little promotion of the severity of micronutrient deficiencies and prevention mechanisms like VAS intake.

A 27 year-old rural resident’s mother stated that

“…Most of the time, the health extension workers advise us on vaccination and child feeding practices, but not on VAS and its importance.”

Another 32 year-old mother also commented that

“…Most of the time I have contact with the health extension workers, but they never told me the severity of vitamin A deficiency and its protective mechanisms. I don't know the importance of VAS or when it is taken.”

A 30 year-old nutrition focal person also noted that

“…The promotion and counseling service for VAS is very low among all health professionals. They did not give equal emphasis to things like vaccination, ANC, and child feeding practices. ”

A 31 year-old urban resident housewife also made this note in a commanding way.

“…Most of the time they (the health extension workers) did not give us information on the services we get; they simply appoint us and bring our children, and we receive any service without having adequate information on it. For example, I don’t know about the benefits of VAS or its schedule. So, the way the service is delivered should be accompanied by adequate information for each service.”

### Workload Among Health Extension Workers

Interviews with the Woreda health office head, the health center head, and the health extension professionals showed that the health extension packages are very vast, which leads to overburden among health extension workers. This consequently led to lower service delivery for the needy community.

A 28 year-old health extension worker commented that

“…We are allowed to work on a lot of activities at a time and to report them timely. Because of this, personally, I place an emphasis on vaccination and Plumy-Nut distribution for moderately and severely malnourished children. To increase the quality and coverage of delivery of all health packages, it is good to classify the packages at least into two or three departments and employ a health extension worker per department.”

A 41 year-old child health and nutrition officer also supported that.

“…The health extension packages are very vast. And they are loaded with routine activities such as vaccination of children, follow-up of malnourished children, family planning, growth monitoring, and communicable disease prevention and control. Because of this, they may not deliver VAS strictly following the schedule. The catchment area for some health posts is also very wide compared to the number of deployed health extension workers.”

## Discussion

According to this research, a lack of information and awareness among parents about the importance of VAS and its schedule is a barrier to VAS uptake among children. Studies conducted in southern Ethiopia [16, 25], Kenya [17], and Ghana [18] support this finding. Other studies have found that media exposure [19, 20] and ownership of a radio or television [26] have a positive impact on VAS coverage, implying that they will raise population awareness. A study conducted in the Amhara region of Ethiopia’s west Gojjam zone found that mothers who believed they understood the HEPs were more likely to visit the health post and obtain the program packages [27]. If mothers are unaware of the significance of VAS and the current timetable, they are less likely to bring their children to receive the service [28]. Maternal health awareness is a key factor in encouraging the adoption of health-supportive behaviors and understanding the significant health effects of VAS [29].

This qualitative finding also showed that, in contrast to vaccinations and other curative services, parents assign low priority to VAS and its schedule despite knowing how important it is. Thus, developing national and local communication strategies will boost mothers’ desire for these essential services for children’s health [30]. Implementing alternative VAS delivery strategies, such as community health days, mass campaigns, and offering health education, will boost community coverage and motivation in addition to the regular service delivery [31].

Low promotion of the VAS scheme and the lack of attention provided to the VAS scheme by health professionals were found to be contributors to low VAS coverage. Instead of focusing on preventive services, health workers typically focus on curative services and addressing current health complaints. This might be the primary factor in the lack of attention aimed at raising awareness of those preventive services [32]. The other explanation might be that some health extension workers weren't adequately trained for all tasks during pre-service education [33]. Workload and burnout, decreased community acceptance of health services, slow career advancement, a lack of a transfer policy, and insufficient financial rewards were some of the factors that demotivated health extension workers (HEWs), which ultimately compromised service delivery [34–36].

One of the reasons for the low coverage of VAS is also the workload of health extension workers, who must handle numerous healthcare packages at once. The Health Extension Program was launched at scale in 2003 with 17 packages under four areas, namely [1]: family health services (maternal and child health, family planning, immunization, adolescent reproductive health, nutrition) [2]; disease prevention and control (HIV/AIDS, TB, malaria, first aid) [3]; hygiene and environmental sanitation (proper and safe excreta disposal, proper and safe solid and liquid waste management, water supply safety measures, food hygiene and safety measures, a healthy home environment, arthropods and rodent control, personal hygiene); and [4] health education and communication [37,38]. Most of the time, there are not enough health extension workers assigned to provide those services, and they lack the time to visit every household to promote health and provide quality care services [36]. Due to their workload, they might prioritize providing curative services like distributing Plumpy Nuts and treating infectious diseases like malaria and diarrhea, and they also intend to leave their jobs [35]. A study done in the Amhara region of Ethiopia indicated that mothers who had frequent household visits by the HEWs were more likely to visit health posts [20]. Even though women’s development armies are assigned to each Keeble’s to support health extension workers in delivering some of the services and promoting the community for service utilization, they are not adequately trained and strengthened [39].

Although not supported in this research, a qualitative finding from a study conducted in Nigeria revealed that the father’s disapproval, unspecified reasons, and peer advice were the barriers to low coverage of VAS [40]. Another research confirmed that one of the factors influencing children’s uptake of VAS is the educational level of their parents [20].

### Strengths and Limitations

In order to uncover deeper insights, the research used qualitative designs to examine VAS from the perspectives of parents and health professionals. The subjects’ varied socio-demographic backgrounds allowed for the exploration of a range of opinions and viewpoints. As a drawback, social-desirability bias may be observed in the interviewees’ answers.

### Conclusion and Recommendations

The major barriers to the uptake of VAS were both client- and supply-side. Awareness about the importance of VAS and its schedule should be promoted among mothers. Health workers should receive refresher training in order to raise their level of concern regarding the significance of the VAS and its schedule. Additionally, expanding the number of facilities or concentrating outreach efforts in farther-flung areas may increase children’s use of VAS. In order to improve service quality and scope, it is also essential to develop strategies that focus on the work environment, increasing the number of health extension workers per cluster and dividing the 17 health extension packages into departments.
